# Unusual neoplasm on the hard palate of a child: a case report

**DOI:** 10.1186/s13256-017-1321-4

**Published:** 2017-06-02

**Authors:** Mathangi Kumar, Shruthi Acharya, Kanthilatha Pai, Vijay Kumar, Sundeep P Thotan

**Affiliations:** 1 Department of Oral Medicine and Radiology, Manipal College of Dental Sciences, Manipal University, Manipal, 576104 Karnataka India; 2 Department of Pathology, Kasturba Medical College, Manipal University, Manipal, 576104 Karnataka India; 3 Department of Pediatric Surgery, Kasturba Medical College, Manipal University, Manipal, 576104 Karnataka India

**Keywords:** Salivary gland tumor, Pediatric, Oral cavity, Hemangioma, Myoepithelioma

## Abstract

**Background:**

Myoepitheliomas account for less than 1% of salivary gland tumors. They mostly affect the parotid glands of adults during the third to fifth decades.

**Case presentation:**

A 10-year-old Indian boy reported a small swelling in the roof of his mouth of 10 days’ duration. History revealed that the lesion was painless and not associated with bleeding or pus discharge. On examination, a purplish well-circumscribed growth was noted on his posterior hard palate. Magnetic resonance imaging was suggestive of a well-encapsulated hemangioma. An excisional biopsy was performed and histopathology along with immunohistochemistry analysis showed that the lesion was a spindle cell variant of benign myoepithelioma.

**Conclusion:**

Palatal myoepitheliomas are rare and their occurrence in young individuals is rarer.

## Background

Myoepitheliomas are rare benign salivary gland neoplasms accounting for less than 1% of salivary gland tumors. They commonly affect the parotid glands and rarely the minor salivary glands, of which the palate is the most common location. Myoepithelioma affects adults between the third and fifth decades, with no gender predilection. To date, to the best of our knowledge, only seven cases of myoepithelioma of the palate occurring in children and adolescents have been reported in the literature. Here we report a case of benign myoepithelioma of the hard palate in a 10-year-old boy to attest the rarity of this tumor.

## Case presentation

**Fig. 1:**
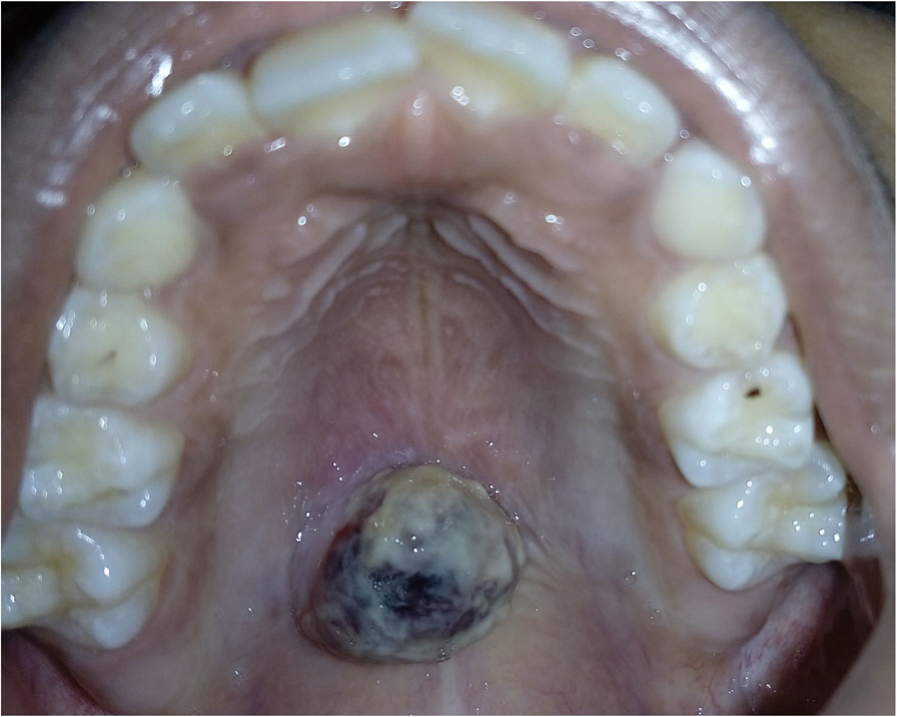
A well-circumscribed palatal lesion along the midline

**Fig. 2:**
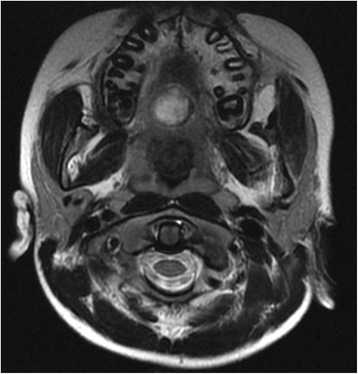
Contrast-enhanced magnetic resonance imaging showing the well-encapsulated lesion with thinning of the floor of the nasal cavity

A 10-year-old healthy Indian boy presented to the Department of Oral Medicine and Radiology with a complaint of a small swelling in the roof of his mouth. His parents reported that the swelling was noticed 10 days earlier and he had no prior complaints. The onset was spontaneous with no history of trauma due to any cause. It was associated with minor bleeding and mild pain on chewing food. He did not report any nasal block or epiphora. His past medical history (immunized for age), social history, family history, and environmental history were noncontributory. General examination revealed normal vital signs (afebrile, heart rate of 100 beats/minute) with no signs of pallor, cyanosis, or icterus. His height was 108 cm and his weight was 18 kg. He was conscious, cooperative, and well oriented to time, place, and person. There were no demonstrable neurological deficits. Intraoral examination revealed a well-circumscribed growth along the midline on the posterior aspect of his hard palate, approximately 2 cm in diameter, dark purple in color with overlying whitish-yellow pseudomembrane (Fig. 1). The surface over the growth appeared slightly irregular. On palpation the lesion was sessile and soft-to-firm in consistency with tenderness. There was no bleeding on manipulation and a diascopy test showed mild blanching. Taking his young age and clinical presentation into account, hemangioma/vascular malformation, pyogenic granuloma, and angina bullosa hemorrhagica were considered for clinical differential diagnoses. Contrast-enhanced Magnetic resonance imaging revealed that the oral lesion was well encapsulated with thinning of the floor of his nasal cavity (Fig. 2), which indicated the possibility of a hemangioma. Considering the clinical and radiological investigatory findings, the differential diagnoses that were taken into account were encapsulated vascular lesions and palatal minor salivary gland tumors. His preoperative laboratory values were all within the normal range: hemoglobin 10 g/dl; hematocrit 32%; mean corpuscular hemoglobin (MCH) 17.6 pg; mean corpuscular hemoglobin concentration (MCHC) 31.2 g/dl; mean corpuscular volume (MCV) 56.3 fl; platelet count 285×10^3^/μl; red blood cell (RBC) count 5.68×10^6^/μl; total white blood cell (WBC) count 14×10^3^/μl; activated partial thromboplastin time (APTT) 28.5 seconds; serum urea 18 mg/dl; and serum creatinine 0.5 mg/dl. Serological tests were non-reactive for human immunodeficiency virus and hepatitis B. Following this he was posted for elective surgical excision of the lesion. Complete removal of the growth was done under general anesthesia and the specimen was sent for histopathological examination.

**Fig. 3:**
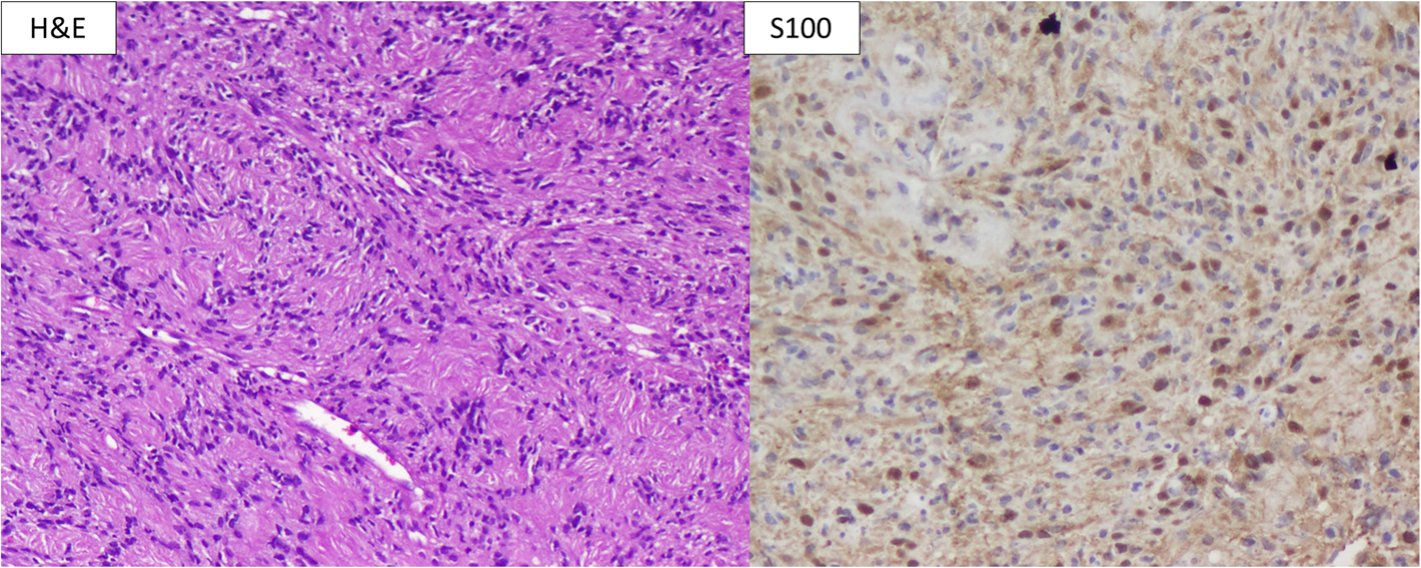
Photomicrograph (hematoxylin and eosin ×200). *Left panel* shows bland spindle-shaped cells with oval vesicular nuclei and cytoplasmic processes arranged in sheets and swirls. *Right panel* shows nuclear and cytoplasmic positivity for S100 on immunohistochemical staining

Histopathological examination revealed a tumor with surface ulceration and inflammation, composed of bland spindle-shaped cells that had ovoid-shaped to spindle-shaped tumor cells arranged in a fascicular pattern and swirls with intervening pale mucoid to myxoid stroma. No areas of increased mitosis, necrosis, or pleomorphism were noted. As the tumor tissue was received in multiple bits, the marginal clearance could not be commented upon. However, the tumor lacked features such as infiltrative growth pattern, necrosis, or perineural invasion owing to which a diagnosis of benign myoepithelioma was considered. Differentiating features between benign and malignant counterparts of myoepithelioma are cytological atypia and mitosis. The tumor cells on immunohistochemistry stained positive with S100, CK5/6, and p63 (Fig. 3). A diagnosis of palatal myoepithelioma (benign spindle cell variant) was made based on the above features.

His postoperative recovery was uneventful with complete healing of the palatal area and no clinical evidence of recurrence (7 months postoperation). He was advised to have clinical/ radiological follow-ups on an annual basis.

## Discussion

Myoepithelioma comprises 1 to 1.5% of all salivary gland neoplasms. It was considered a variant of pleomorphic adenoma, but now it is recognized as a distinct entity. The parotid gland is commonly affected followed by the hard palate [1]. Only 21% of myoepitheliomas are reported to occur on the palate [2]. The World Health Organization characterizes myoepitheliomas as showing a more aggressive growth pattern than pleomorphic adenomas [3]. Myoepitheliomas may be benign or malignant and are relatively a rare occurrence. Most cases of salivary gland myoepitheliomas have been reported in middle aged adults with no definite sex predilection. On the other hand, there are only a few cases of palatal myoepitheliomas reported in the literature in children and adolescents. Reports of palatal myoepithelioma in the literature are summarized in Table 1.

**Table 1:** Reports of palatal myoepithelioma in the literature

Authors	Age (years)	Gender	Histological type
Kahn and Schoub (1973) [5]	17	Female	Plasmacytoid
Nesland *et al*. (1981) [6]	18	Female	Plasmacytoid
Lins and Gnepp (1986) [7]	8	Female	Plasmacytoid
Arkuszewski *et al*. (2005) [8]	12	Male	Plasmacytoid
Nwoku *et al*. (2005) [9]	11	Male	Plasmacytoid
Perez *et al*. (2007) [10]	13	Male	Plasmacytoid
Santos *et al*. (2011) [11]	15	Male	Plasmacytoid
Our case (2017)	10	Male	Spindle cell type

On histologic examination, myoepitheliomas are of four morphological types: spindle cell (most common), epithelioid, plasmacytoid, and clear cell (least common). The most common histopathological type described in palatal myoepithelioma is of the plasmacytoid type, while our case revealed a spindle cell morphology. The spindle cell variant of myoepithelioma is not known to behave differently when compared to other morphological subtypes of myoepithelioma. On immunohistochemical examination, the neoplastic cells stain positive with cytokeratin (AE1/AE3, CK5/6, CK7) and variably positive for S100, smooth muscle actin (SMA), p63 protein, and glial fibrillary acidic protein (GFAP). Usually, a combination of a keratin in conjunction with the detection of S100, vimentin, and/or a myogenic marker is required for confirmation of the diagnosis of a myoepithelioma. The present case showed positivity with S100 and p63, thus confirming the diagnosis of myoepithelioma.

Although minor salivary gland tumors present clinically as a dome-shaped swelling on the palate, there may be altered surface texture of the lesion, as in the present case, which may be trauma induced because the oral cavity is subjected to varying temperatures, pH, and different textures of food substances and beverages apart from the masticatory forces. The unusualness of the present case lies in the fact that the tumor that presented on the hard palate of a young child resembled a hemangioma but was found to be a spindle cell type of myoepithelioma, which is a very rare entity. Surgical excision of myoepithelial tumors with adequate margins decreases the chances for recurrence. Further, the reported recurrence rate is the same as that of pleomorphic adenoma (40 to 65%) [4]. The prognosis for benign myoepitheliomas is generally favorable, provided patients undergo regular follow-up examinations to eliminate local recurrence [2].

## Conclusions

Benign salivary gland neoplasms like myoepitheliomas should be considered in the differential diagnoses of palatal lesions. Although minor salivary gland tumors present as dome-shaped, smooth-surfaced masses on the palate, it should be borne in mind that there can be alteration in the surface characteristics; thus mimicking other vascular and reactive lesions.
